# Food hygienic practices and associated factors among street food vendors in Bishoftu town, central Ethiopia

**DOI:** 10.1016/j.heliyon.2024.e40938

**Published:** 2024-12-05

**Authors:** Firaol Girma, Tamiru Yazew, Dinaol Bedada, Agama Daba, Chala G. Kuyu

**Affiliations:** a Department of Public Health, College of Health Sciences, Salale University, Fitche, Ethiopia; b Department of Food and Nutritional Sciences, Faculty of Agriculture, Wollega University, Nekemte, Ethiopia; c Department of Postharvest Management, Jimma University College of Agriculture and Veterinary Medicine, Jimma, Ethiopia

## Abstract

**Background:**

Poor food hygiene practices in developing nations like Ethiopia pose a significant public health threat by increasing the likelihood of food-borne infections. In the study area, there is a lack of comprensive information regarding the hygienic standards and associated factors influcncing the food hygienic practices of street food vendors. Therefore, this study aims to assess food hygienic practices and related variables among street food vendors in Bishoftu town, central Ethiopia.

**Method:**

A cross-sectional study was conducted from June 16, 2023 to August 16, 2023, involving 210 street food vendors selected through a simple random sampling technique. Data were collected through face-to-face interviews and analysed using Statistical Package for the Social Sciences (SPSS) version 21. Descriptive statistics like mean, frequency, and percentages were used. Logistic regression analysis was performed to determine the significance of the relationship between each independent and dependent variables. The strength of the association was calculated using adjusted odds ratio (AOR) and the 95 % CI and a p-value of 0.05 was considered statistically significant.

**Results:**

The finding of this study revealed that about 64.8 % and 35.2 % of the street food vendors had good and poor food hygiene practices, respectively. Variables such as being female [AOR = 0.191, 95 % CI (0.043, 0.840)], having rich experience in food vending [AOR = 5.793, 95%CI (1.419, 23.649)], maintaining a clean environment [AOR = 2.860, 95%CI (1.484, 5.512)], and possessing knowledge of food hygiene [AOR = 2.726, 95 % CI (1.317, 5.643)] were significantly associated with the food hygiene practices of street vendors.

**Conclusion:**

The level of food hygiene practice observed among street food vendors in the study area was lower compared to other study findings conducted in different regions of Ethiopia. Significant factors influencing these hygiene practices were sex, food vending experience, environment, and knowledge. Therefore, current national regulations must be revised to address the existing gaps and enhance food safety procedures for street food vendors nationwide.

## 1 Introduction

Around the world, approximately 2.5 billion people consume street food every day [1]. The Centers for Disease Control and Prevention (CDC) reported about 737 foodborne disease outbreaks, resulting in 52,011 cases, with 33 % of these outbreaks linked to food from street vendors [2]. Every year, 325,000 people are sent to the hospital and more than 420,000 deaths occur yearly [3].

Based on reports from the Food and Agricultural Organization (FAO), one in ten people worldwide experiences food poisoning each year. Comparable to the impact of HIV/AIDS or malaria, the burden of foodborne diseases on public health is significant [1]. Developing nations bear almost this entire load (98 %), and the majority of it (97 %) is caused by foodborne pathogens including viruses, bacteria, and parasites [4]. Eating food tainted with germs, viruses, parasites, or chemical contaminants such as heavy metals can result in food-borne diseases. Health issues that are both acute and chronic can result from parasitic illnesses that are transmitted through food. Parasites are thought to affect 48.4 million people annually, and 48 % of those infections are spread via food [5]. Children under five are more likely to carry 40 % of the burden of foodborne diseases [6]. Furthermore, one in six diarrheal deaths in this age group is attributed to contaminated food [1]. One in six cases of diarrheal deaths in this age range was caused by contaminated food [1].

Poor food hygiene practices in developing countries, particularly in Africa, contribute to morbidity, food-borne diseases, and digestive disorders during food preparation, handling, and storage [7,8]. In Africa, intestinal parasite infections result in more than 200,000 fatalities, which are made worse by poor food hygiene practices [9]. More than 91 million foodborne disease cases and 137,000 deaths also occurred in Sub-Saharan Africa [10]. As they contribute to public health and social problems, food handling practices and safety are the most significant health and safety challenges in developing countries [11].

Foods vended in the street usually have a high chance of getting contaminated by food vendors; this most often dependes on the health status of the food handler, the vending location, and their hygiene behaviors and practices [11]. As a result, the food sold on the street foods were contaminated with food-borne pathogenic bacteria, which might threaten the general public [8,12,13]. With the rise in reports of health issues linked to poor food hygiene, it is crucial to understand the hygiene measures used by food vendors.

According to the World Health Organization (WHO), five keys to safer food are important in preventing foodborne diseases. The key food safety practices include; keeping food surfaces clean, promptly washing utensils and dishes, separating raw and cooked foods, thoroughly cooking food to the correct temperature, maintaining safe temperatures for serving and storage, and using safe water and raw materials [14]. Many developing countries worldwide have weak street food legislation and regulations. In India, although only about one-third of street food vendors are registered, many still fail to follow essential food safety practices. These include wearing aprons, access to tap water, and ensuring proper food storage [15].

### 1.1 Food hygiene legislation in Ethiopia

Having an effective food safety system is vital for protecting the public from unsafe food and adverse health effects, such as foodborne illnesses [16]. The 2018 Food and Nutrition Policy (FNP) in Ethiopia has recognized the importance of food safety and nutrition as a responsibility of the government at the national level [17]. The Ethiopia food safety stytem lacks the organization and expansion compared to other developed countries [18]. Moreover, challenges such as urbanization, environmental issues, and food hygiene concerns place additional strain on the country's food systems, negatively affecting the nutrition value of food and its safety.

In Ethiopia, food safety regulations do not include traditional markets, which are frequented by numerous Ethiopians for buying and selling food [19]. The regulations governing the enforcement of food safety controls have numerous unfilled gaps and are occasionally outdated, presenting an opportunity to introduce updated strategies [20]. The current strategy for food safety and control primarily targets agricultural produce. However, there is a need for a more cohesive approach to link food safety with consumption.

Despite being a pioneer in prioritizing food safety in Africa, Ethiopia will continue to face significant challenges in managing food safety within its informal and transitioning food systems. Effective change will require a strong demand from consumers for safer food and the ability of vendors to meet these expectations [21]. Regrettably, many low- and middle-income countries (LMICs) have struggled to effectively engage consumers in food safety matters to provide safer food services [22]. Furthermore, there is a lack of comprehensive understanding of the practices of food suppliers' and consumers’ food choice to food safety in these countries [21]. Thus, training can enhance understanding [23], yet thorough assessment is essential.

Although there are numerous advantages, existing regulatory definitions of "food trade" fail to consider traditional markets, leading to insufficient public funding for market infrastructure and inadequate monitoring of foodborne diseases [24]. The absence of robust health and safety standards, along with enforcement actions such as vendor licensing and regulations on food handling, further exacerbates the issue [25]. In Ethiopia, there are food items and food products that have yet to have mandatory standards set in place [26,27].

However, the prevalence of food-borne diseases in Ethiopia ranges from 14.5 to 57.8 % [[28], [29], [30], [31], [32], [33]], there are estimated to be 13 deaths per year from foodborne disease per 100,000 people [10], and its level ranges from 50.9 % to 80 % [8,34]. In addition, a study conducted in Ethiopia, street-vended foods showed a high level of bacterial contamination [35]. Nearly 70 % of diarrheal illnesses are also associated with the consumption of contaminated food [36]. In the Jimma town of Oromia region, the prevalence of intestinal parasites among street food handlers was 39.2 %, and the prevalence of enteric bacterial pathogens was 8.85 % [33], and this could be due to poor knowledge and negative attitude during food handling, preparation and serving [37].

Furthermore, a research conducted in northwest Ethiopia revealed that approximately 50 % of the participants lacked adequate knowledge and practice in food hygiene [34]. Additionally, a research carried out in the northeastern region of Ethiopia revealed that approximately 57.2 % of street food vendors exhibited inadequate food and safety practices [38]. The study finding from Ethiopia also reported that the level of poor food hygiene practices food vendors was 41.1 % [39].

Factors that indicate good hygienic practices among street food vendors include gender, professional background, educational level, monthly income, food safety training experience, medical examinations, access to handwashing amenities, and a strong understanding of food safety [[40], [41], [42]].

Generally, poor personal hygiene, the mixing of raw and processed foods, insufficient cooking, and incorrect food storage are all potential sources of food contamination for street food vendors. Many consumers frequently indulge in street-vended foods in towns due to their easy accessibility, affordability, and quick availability [43]. However, the data regarding the level of hygienic practices and its predictors among the study particpiants in the study area was limited. Many stakeholders within the food value chain in conventional market settings are also deficient in knowledge and training pertaining to efficient food risk management systems. Hence, this study assessed food hygiene practices and its ifnlucning factors among street food vendors of Bishoftu town, central Ethiopia, aiming to shed light on their current practices and areas that could benefit from improvement.

## 2 Method

### 2.1 Study design and setting

The study was conducted in Bishoftu town, central Ethiopia from June 16 to August 16, 2023. Bishoftu town is a resort town located 47.9 km from Addis Ababa. According to 2021 estimates, Bishoftu town has 93,631 males and 103,926 females, with a total population of 197,557. The town is located at a latitude of 8°45 ′ N & 38°59 ′ E, longitude of 8.75°N &38.983°E, and elevation of 1920 m above sea level [27]. Bishoftu town has six health centers and twohospitals (one governmental and one non-governmental). The Bishoftu town Health Office, Culture and Tourism Office, and trade office have reported a lack of data on street food vendors, despite the high population growth, urbanization, and street food marketing in the town.

### 2.2 Sampling and data collection methods

All street food vendors found in Bishoftu town were considered as the source population while those randomly selected were the study population. All food vendors who have been vending street foods on the street for more than six months were included in the study. The study did not include individuals who did not direct contact with food sold packaged foods, or who were mobile street food vendors were not included in the study. To determine the sample size, a single population proportion formula with assumptions like a 95 % confidence level, a 49.4 % prevalence of food handling practices [34], and a 5 % margin of error (d) were considered. Thus, the sample size was 384. A correction formula was used since the total population of the study area was less than 10,000, and the final required sample size for this study was 219.

In this study, the dependent variable was the food hygienic practices, which has two categorical variables asnd coded as (0 = poor food hygienic practices and 1 = good food hygienic practice). Socio-demmographic factors; participants’ age, educational staus, sex, marital status, and work experience and knowledge concerning the food vending were used as predictors.

The simple random sampling technique was used to get the required sample size the registered food vendors. The street food vendors were selected using computer generated simple random method. If more than one food handler is found in the food vending units, lottery method was used to choose one of them. Questionnaires were developed, and the data were collected by three trained BSc in nutrition professionals and supervised by one MSc in nutrition.

The tools were developed based on several previously published articles [39,41,42]. For the knowledge, 15 related questionnaires were used, and each question had one point for ‘Yes’ and zero point for ‘No’. Finnaly, respondents who scored below the mean were classified to have e poor knowledge, whereas those scored at or above the mean were classified as having good knowledge. Similary, 12 questions were used to assess food hygienic practices. Each question had 0 for ‘No’ response and 1 for ‘Yes’. Thus, the respondents who scored less than the mean had poor food practices and those who scored greater than or equal to the mean had good practices.

The questionnaire was first developed in English and translated into Afaan Oromoo. Then, it retranslated into the English language to ensure its uniformity. To ensure the quality of the data, training, and pre-test (5 %) were conducted. The Cronbach alpha was computed to measure the reliability and validity (internal consistency) between items in the scale for measuring practice towards street food vending and was found to be 0.82. Since the value of Cronbach alpha is greater than 0.7, the reliability of tools were good.

### 2.3 Statistical analysis

The data were checked for completeness in Epidata and then exported to SPSS version 21. Descriptive statistics, including frequencies, mean, standard deviations, and percentages, were calculated. Cross-tabulations and bivariate logistic analysis were used to evaluate the association of independent variables with the outcome variable. Variables with a p-value ≤0.25 were included in a multivariable logistic regression model to control for confounders. Multivariable logistic regression identified the association of independent variables (e.g., age, sex, salary, and experience, environment, and knowledge) with the dependent variable. The strength of association was determined using an adjusted odds ratio with a 95 % confidence interval, considering a p-value of less than 0.05 as statistically significant.

## 3 Results

### 3.1 Sociodemographic characteristics of participants

Among 210 respondents, nearly 142 (67.6 %) comprised individuals aged 18–30. The largest portion of the participants, 88(41.9 %) attended secondary school, 61(29 %) attended primary school and 57(27.1 %) attended higher education. About 97(46.2 %) and 92(43.8 %) participants were married and single, respectively. A significant portion, 83(39.5 %) study subjects reported less than two years of professional experience (Table 1).

**Table 1:** Sociodemographic features of street food sellers in Bishoftu, central Ethiopia.

Variables	Variable categories	Frequency (n)	Percent (%)
Age of the participants	18–30 years	142	67.6
	31–40 years	47	22.4
	Above 41 years	21	10
Participants sex	Male	14	6.7
	Female	196	93.3
The educational level of the participant	No formal education	4	1.9
	Primary	61	29.0
	Secondary	88	41.9
	Diploma and above	57	27.1
Marital status of the participants	Single	92	43.8
	Married	97	46.2
	Divorced	13	6.2
	Widowed	8	3.8
Work experience of the participant	Less than 2 years	83	39.5
	2–5 years	100	47.6
	More than 6 years	27	12.9

### 3.2 Hygienic practice of street food vendors

Table 2 depicts that about 207 (98.6 %), 136 (64.82 %), 146 (69.5 %) of the street food sellers did not use gloves, hair caps, and apron, respectively. Of all the street food vendors, about 194 (92.4 %) participants used soap for hand washing, 173 (82.4 %) trimmed their nails, 125 (59.5 %) removed jewelry, 155 (73.8 %) washed their bodies regularly. Besides, Food hygiene practices were observed to be good among 136 (64.8 %), while 74 (35.2 %) participants (35.2 %) exhibited poor practices.

**Table 2:** Personal Hygienic practice of the study particpants in Bishoftu, centeral Ethiopia.

Variables	Categories	Frequency (n)	Percent (%)
Gloves usage	No	207	98.6
	Yes	3	1.4
Hair cover usage	No	136	64.8
	Yes	74	35.2
Apron Usage	No	146	69.5
	Yes	64	30.5
Using gear is important	No	0	0.0
	Yes	210	100.0
Do you apply appropriate temperature during food preparation and storage?	No	75	35.7
	Yes	135	64.3
Do you use proper methods for cross- contamination prevention?	No	56	26.7
	Yes	154	73.3
Do you use appropriate cleaning practices?	No	72	34.3
	Yes	138	65.7
Do you wash your hands before touching foods	No	0	0.0
	Yes	210	100.0
Do you use soap for hand washing	No	16	7.6
	Yes	194	92.4
Do you trim your nails and make sure they are clean	No	37	17.6
	Yes	173	82.4
Do you remove any jewelry before handling food	No	85	40.5
	Yes	125	59.5
Do you wash your body regularly	No	55	26.2
	Yes	155	73.8
Overall food hygienic practices	Poor food hygienic practice	74	35.2
	Good food hygienic practice	136	64.8

### 3.3 Environmental hygienic practices

Most, 204 (97.1 %) street food vendors cleaned their utensils in a bowl of water and 167 (79.5 %) changed the water every hour (Table 3). About 7(3.3 %) of the participants disposed of their refuse and wastes in an open field. Moreover, about 118 (56.2 %) of the participants had a clean environment for street food processing.

**Table 3:** Environmental Hygienic practices of the study particpants in Bishoftu, central Ethiopia.

Variables	Categories	Frequency (n)	Percent (%)
If you wash in a bowl of water how often it is changed	Within 30 min	26	12.4
	Every hour	167	79.5
	Every 6 h	17	8.1
Where do you dispose of your refuses	In a dustbin nearby	156	74.3
	In a dustbin far away	47	22.4
	On the open field	7	3.3
How often is the refusal bin emptied	Whenever it is full	89	42.4
	By the close of the day	112	53.3
	Every other day	2	1
	No dustbin	7	3.3
Do you cover your refuse	No	17	8.1
	Yes	193	91.9
Do you carry out daily cleaning of your premises	No	3	1.5
	Yes	207	98.5
Clean vending environment	No	92	43.8
	Yes	118	56.2
Availability of portable water	No	80	38.1
	Yes	130	61.9
Availability of storage and heating equipments	No	114	54.3
	Yes	96	45.7
Access to electricity	No	87	41.4
	Yes	123	58.6
	Yes	114	54.3

### 3.4 Knowledge towards hygienic practices

Hygienic practices among street food vendors in Bishoftu were assessed, revealing a significant knowledge gap. Table 4 shows that 80 % (168 vendors) demonstrated poor understanding of proper hygiene, while only 20 % (42 vendors) exhibited adequate knowledge.

**Table 4:** Knowledge towards hygienic practices of the study participants in Bishoftu, central Ethiopia.

Variables		Categories	
		Yes, n(%)	No, (%)
Washing hands regularly after work is a vital aspect of maintaining personal hygiene.		210(100)	0(0)
Proper hand-washing minimizes the chance of food contamination		210(100)	0(0)
Water alone is insufficient for effective hand cleaning		189(90)	21(10)
Maintaining personal hygiene includes wearing masks, gloves, and hair coverings where appropriate		172(81.9)	38(18.1)
After hand-washing, vendors should refrain from touching their hair		210(100)	0(0)
Consuming food at work increases the risk of foodborne contamination		8(3.8)	202(96.2)
Maintaining clean equipment and proper handling practices minimize contamination risks		209(99.5)	1(0.5)
Wearing gloves helps reduce the risk of food contamination		177(84.3)	33(15.7)
Damaged gloves should be replaced with new ones		205(97.6)	5(2.4)
Proper equipment minimizes food contamination after cleaning		210(100)	0(0)
Cleaning equipment using hot water reduces the risk of contamination		210(100)	0(0)
Contamination can be reduced by separating areas of cocked and uncocked		206(98.1)	4(1.9)
Changes in color or odor can indicate food contamination		210(100)	0(0)
Reusing oil is unsafe to health		59(28.1)	151(71.9)
Improper reheating can lead to food contamination		35(16.7)	175(83.3)
Over all Knowledge status	Poor Knowledge	168 (80 %)	
	Good knowledge	42 (20 %)	

### 3.5 Factors associated with hygiene practices

According to the finding of this study, there were variables like vending clean environment, work experience, being female and knowledge of food hygiene which were significantly related with hygienic issues (Table 5).

**Table 5:** Binary and multivariate logistic regression analysis of associated factors with food hygiene practices among street food vendors in Bishoftu, central Ethiopia.

Variables	Categories	The sanitation practices of street food vendors		COR(95%CI)	AOR(95%CI)
		Good (%)	Poor (%)		
Age	18–30years	62(43.7)	80(56.3)	1	1
	31–40years	26(55.3)	21(44.7)	1.598(0.823, 3.103)	0.365(0.142, 0.940)
	>41years	14(66.7)	7(33.3)	2.581(0.982, 6.780)	0.521(0.139, 1.951)
Sex	Male	9(64.3)	5(35.7)	1	1
	Female	93(47.4)	103(52.6)	0.502(0.162, 1.551)	0.191(0.043, 0.840)**∗∗**
Marital status	Single	33(35.9)	59(64.1)	1	1
	Married	56(57.7)	41(42.3)	2.442(1.359, 4.389)**∗**	1.912(0.787, 4.648)
	Divorced	7(53.8)	6(46.2)	2.086(0.647, 6.725)	1.695(0.387, 7.412)
	Widowed	6(75.0)	2(25.0)	5.364(1.024, 28.098)**∗**	2.634(0.260, 26.730)
Work experience	≤2years	28(33.7)	55(66.3)	1	1
	2–5years	52(52.0)	48(48.0)	2.128(1.167, 3.880)**∗**	1.519(0.702, 3.287)
	>5 years	22(81.5)	5(18.5)	8.643(2.958,25.257)**∗**	5.793(1.419, 23.649)**∗∗**
Utensil washing water changing frequency	Within 30 min	17(65.4)	9(34.6)	1	1
	Every hour	80(47.9)	87(52.1)	0.487(0.205, 1.154)	1.015(0.356, 2.894)
	Every 6 h	5(29.4)	12(70.6)	0.221(0.059, 0.825)	0.460(0.091, 2.313)
Cover refuses	No	3(17.6)	14(82.4)	1	1
	Yes	99(51.3)	94(48.7)	4.915(1.369, 17.650)**∗**	2.236(0.537, 9.300)
Clean vending environment	No	30(32.6)	62(67.4)	1	1
	Yes	72(61.0)	46(39.0)	3.235(1.826, 5.730)**∗**	2.860(1.484, 5.512)**∗∗**
Food hygiene Knowledge	Poor	24(30.0)	56(70.0)	1	1
	Good	78(60.0)	52(40.0)	3.500(1.934, 6.334)**∗**	2.726(1.317, 5.643)**∗∗**

## 4 Discussion

This study finding revealed that about 35.2 % exhibited poor hygiene practices, a lower percentage than reported in studies from Gondar, Ethiopia [44], and other Ethiopian regions [38,[45], [46], [47]]. Likewise, the study finding is less than that of studies carried out in different regions Ethiopia [[39], [40], [41],^48^], and Ghana [49], India [50] and Nigeria [51]. Furthermore, a significant association emerged between vendor gender and hygiene practices, with female vendors displaying poorer hygiene than their male counterparts. This study finding aligns with the previous research in Ethiopia [40,44,52].

Multivariate logistic regression revealed a positive correlation between vendor experience and adherence to hygienic practices. The result of the present study is aligning with research from Ethiopia [39,40,47,52,53], Nigeria [54], and South Africa [55]. This suggests that experience fosters better hygiene practices. As vendors gain more experience, their ability to maintain effective hygiene practices also increases. This is attributed to the knowledge and skills acquired over years of practice in the food vending industry.

Similarly, vendors operating in clean environments were more likely to maintain good hygiene, supporting findings from Ethiopia [41,46] and South Africa [55]. Cleanliness, therefore, appears to be a significant factor in promoting food safety practices, aligning with research indicating that improved environmental conditions can increase awareness of food safety standards [15].

Furthermore, study demonstrates a strong link between knowledge of food hygiene and the adoption of safe practices among street food vendors; those with better knowledge exhibited better hygiene. Different studies from Ethiopia [39,40,43,47] and Nigeria [51] also reported the vital role of education in promoting food safety among street vendors. A solid understanding of food hygiene principles significantly influences vendor practices and their ability to meet consumer expectations for safe food [39,40]. This study is also consistent with other study conducted in low- and middle-income countries [42,48].

## 5 Limitations of the study

This cross-sectional study, conducted over a limited timeframe and with a small sample size, could not establish definitive cause-and-effect relationships between predictor and outcome variables. Potential recall bias during interviews and the lack of a standardized definition of food hygiene practices (due to the absence of national guidelines) may also limit the generalizability of the findings.

## 6 Conclusion

This study reveals a higher prevalence of poor food hygiene among street food vendors in the study area than previously reported elsewhere in the country. Factors significantly associated with food hygiene practices were the vendor sex, experience, environmental cleanliness, and knowledge. The study highlights a lack of active involvement from various sectors despite existing coordination plans for street food vendor activities, leading to insufficient attention towards this group. To address these issues, stakeholder collaboration is recommended for strengthening public health policies and legislations to ensure safe practices and promote a safer, healthier society.

## CRediT authorship contribution statement

**Firaol Girma:** Writing – review & editing, Writing – original draft, Funding acquisition, Formal analysis, Data curation, Conceptualization. **Tamiru Yazew:** Writing – review & editing, Writing – original draft, Visualization, Validation, Supervision, Software, Methodology, Investigation, Funding acquisition, Formal analysis, Data curation, Conceptualization. **Dinaol Bedada:** Writing – review & editing, Writing – original draft, Funding acquisition, Formal analysis, Data curation, Conceptualization. **Agama Daba:** Writing – review & editing, Writing – original draft, Supervision, Resources, Methodology, Funding acquisition, Formal analysis, Data curation, Conceptualization. **Chala G. Kuyu:** Writing – review & editing, Writing – original draft, Software, Methodology, Formal analysis, Data curation, Conceptualization.

## Data availability statement

Data will be made available on request.

## Ethical clearance

The Institutional Review Board (IRB) of Salale University approved all procedures (Reference RRC: No/SLU/241/2023). The university communicated officially with local authorities regarding the study. Participants were thoroughly and transparently informed about the study's procedures and their right to withdraw at any point. Written informed consent was obtained from each participant, and confidentiality was maintained throughout the study.

## Funding statement

This research did not receive any specific grant from funding agencies in the public, commercial, or not-for-profit sectors.

## Declaration of competing interest

The authors declare no conflict of interest.

## References

[1] World Health Organization and Food and Agricultural Organization (2022).

[2] Emmanuel O.I., Ibe Sally N.O., Emmanuel N., Sule O.C. (2019). Knowledge and practice of food hygiene among food vendors in ihiagwa, owerri west local government area, imo state. Texila International Journal of Public Health.

[3] World Health Organization (2015).

[4] Havelaar A.H., Martyn D.K., Paul R.T., Herman J.G., Tine H., Robin J.L., Nicolas P. (2015). World Health Organization global estimates and regional comparisons of the burden of foodborne disease in 2010. PLoS Med..

[5] Centers for Disease Control and Prevention (CDC) (2019). https://www.cdc.gov/parasites/food.html.

[6] World Health Organization (WHO) (2015).

[7] Grace D. (2015). Food safety in low and middle-income countries. Int. J. Environ. Res. Publ. Health.

[8] Adimasu A., Bimerew M., Tadesse G., Zemichael G., Tsegaye A. (2016). Bacteriological quality assessment of selected street foods and their public health importance in Gondar Town, North West Ethiopia. Global Vet..

[9] Ayana Z., Yohannis M., Abera Z. (2015). Food-borne bacterial diseases in Ethiopia. Acad J Nutr.

[10] Grace D., Silvia A., Florence K.M., Kristina R., Johanna F.L., Kebede A. (2018).

[11] Fung F., Wang H.S., Menon S. (2018). Food safety in the 21st century. Biomed. J..

[12] Teferi S.C. (2020). A review on food hygiene knowledge, practice and food safety in Ethiopia. Sci J Food Sc Nutr.

[13] Alem K. (2020). Bacterial load assessment of some food items sold in street in woldia town, north-east Ethiopia. J. Pure Appl. Microbiol..

[14] World Health Organization (2006). Five keys to safer food manual. Food safety.

[15] Reddy A.A., Ricart S., Cadman T. (2020). Driving factors of food safety standards in India: learning from street-food vendors' behaviour and attitude. Food Secur..

[16] Faour-Klingbeil D., Ewen C.D. (2020). Prevention and control of foodborne diseases in Middle-East North African countries: review of national control systems. Int. J. Environ. Res. Publ. Health.

[17] Federal Democratic Republic of Ethiopia (FDRE) (2018).

[18] Gemede H.F., Tamiru Y. (2023). Food hygiene knowledge, practices, and associated factors among food handlers in institutional food establishments in nekemte town, western Oromia, Ethiopia: a cross‐sectional study. J. Food Qual..

[19] Jabbar M.A., Samuel A.A. (2010). The Case of Beef, Raw Milk and Local Butter in Ethiopia.

[20] Global Alliance for Improved Nutrition (2022).

[21] Ortega D.L., David L.T. (2017). Demand for food safety in emerging and developing countries: a research agenda for Asia and Sub-Saharan Africa. J. Agribus. Dev. Emerg. Econ..

[22] Jaffee S., Henson S., Unnevehr L., Grace D., Cassou E. (2018).

[23] Parikh P., Aparo N.O., Nordhagen S., De Steur H. (2022). Food safety-related perspectives and practices of consumers and vendors in Ethiopia: a scoping review. Food Res. Int..

[24] Grace D. (2023). Burden of foodborne disease in low-income and middle-income countries and opportunities for scaling food safety interventions. Food Secur..

[25] Tamene A., Habte A., Woldeyohannes D., Afework A., Endale F., Gizachew A., Sulamo D., Tesfaye L., Tagesse M. (2022). Food safety practice and associated factors in public food establishments of Ethiopia: a systematic review and meta-analysis. PLoS One.

[26] Ayalew H., Birhanu A., Asrade B. (2013). Review on food safety system: Ethiopian perspective. Afr. J. Food Sci..

[27] Temesgen M., Abdisa M. (2015). Food standards, food law and regulation system in Ethiopia: a review. Publ. Pol. Adm. Res..

[28] Wegayehu T., Belete T. Tsegaye S., Takele T. (2013). Prevalence of intestinal parasitic infections among highland and lowland dwellers in Gamo area, South Ethiopia. BMC Publ. Health.

[29] Tefera T., Abdissa B., Zeleke M., Teferi E. (2014). Parasitic contamination of fruits and vegetables collected from selected local markets of Jimma Town, Southwest Ethiopia. Int. Sch. Res. Notices.

[30] Alemu A.S., Baraki A.G., Alemayehu M., Yenit M.K. (2019). The prevalence of intestinal parasite infection and associated factors among food handlers in eating and drinking establishments in Chagni Town, Northwest Ethiopia. BMC Res. Notes.

[31] Alemu G., Mohammedaman M., Desta M. Direslgne H. (2019). Parasitic contamination of vegetables marketed in Arba Minch town, southern Ethiopia. BMC Infect. Dis..

[32] Eshetu L., Geletta D.Regea T. (2019). Prevalence of intestinal parasites and its risk factors among food handlers in food services in Nekemte town, west Oromia, Ethiopia. Res. Rep. Trop. Med..

[33] Gemechu T., Teferi E., Tesfaye K., Habtemu J. (2022). Assessment of intestinal parasites, enteric bacterial infections, and antimicrobial susceptibility among street food handlers in Jimma town, southwest Ethiopia. J. Trop. Med..

[34] Azanaw J., Garedew T.E., Hanna D., Samual B., Siraye D. (2022). Food hygiene knowledge, and practices and their associated factors of street food vendors in Gondar city, Northwest Ethiopia, 2021: a cross-sectional study. Heliyon.

[35] Teferi S.C. (2020). Street food safety, types and microbiological quality in Ethiopia: a critical review. Am. J. Appl. Sci..

[36] Tegegne H.A., Phyo H.W.W. (2017). Food safety knowledge, attitude and practices of meat handler in abattoir and retail meat shops of Jigjiga Town, Ethiopia. Journal of preventive medicine and hygiene.

[37] Akabanda F., Eli H.H., James O. (2017). Food safety knowledge, attitudes and practices of institutional food-handlers in Ghana. BMC Publ. Health.

[38] Adem A.Y., Werkneh A.A., Yimer F. (2023). Food hygiene practices and associated factors among street food vendors in northeastern Ethiopia: a cross-sectional study. Glob Soc Welf.

[39] Werkneh A.A., Tewelde M.A., Gebrehiwet T.A., Islam M.A., Belew M.T. (2023). Food safety knowledge, attitude and practices of street food vendors and associated factors in Mekelle city, Northern Ethiopia. Heliyon.

[40] Moges M., Ernest K.R., Ambelu Argaw A. (2024). Sanitary condition and hygienic practice of street food vendors in selected towns of Ethiopia: a cross-sectional study addressing public health concern. Journal of Agriculture and Food Research.

[41] Weldesenbet H.H., Yawukal C.K., Habtamu N., Dube J. (2024). Food hygiene, safety measures, and associated factors among street food vendors in Addis ababa, Ethiopia: implications for intervention activity design and implementation. J. Food Process. Preserv..

[42] Desye B., Tesfaye A.H., Daba C., Berihun G. (2023). Food safety knowledge, attitude, and practice of street food vendors and associated factors in low-and middle-income countries: a Systematic review and Meta-analysis. PLoS One.

[43] ImathIu S. (2017). Street vended foods: potential for improving food and nutrition security or a risk factor for foodborne diseases in developing countries?. Current Research in Nutrition and Food Science Journal.

[44] Azanaw J., Mulat G., Henok D. (2019). Factors associated with food safety practices among food handlers: facility-based cross-sectional study. BMC Res. Notes.

[45] Abdi A.M., Amano A., Abrahim A., Getahun M., Ababor S., Kumie A. (2020). Food hygiene practices and associated factors among food handlers working in food establishments in the Bole Sub City, Addis Ababa, Ethiopia. Risk Manag. Healthc. Pol..

[46] Negassa B., Adane T.A., Girma W., Abriham S.A., Binyam T.S., Berhanu G.D., Girum G.K., Negasa E.S. (2023). Food hygiene practices and associated factors among street food vendors in urban areas of Gedeo Zone, southern Ethiopia. Environ. Health Insights.

[47] Bantie G.M., Woya A.A., Ayalew C.A., Mitiku K.W., Wubetu G.A., Aynalem Z.B., Ayalew A.F., Ayenew G.M., Akenie B.B., Berneh A.A., Wagaye F.E. (2023). Food safety and hygiene practices and the determinants among street vendors during the chain of food production in northwest Ethiopia. Heliyon.

[48] Zenbaba D., Biniyam S., Fikadu N., Girma B., Fikreab D., Daniel A., Vijay K.C. (2022). Food hygiene practices and determinants among food handlers in Ethiopia: a systematic review and meta-analysis. Trop. Med. Health.

[49] Tuglo T.L., Agordoh P.D., Tekpor D., Pan Z., Agbanyo G., Chu M. (2021). Food safety knowledge, attitude, and hygiene practices of street-cooked food handlers in North Dayi District, Ghana. Environ. Health Prev. Med..

[50] Adane M., Teka B., Gismu Y., Halefom G., Ademe M. (2018). Food hygiene and safety measures among food handlers in street food shops and food establishments of Dessie town, Ethiopia: a community-based cross-sectional study. PLoS One.

[51] Iwu A.C., Uwakwe K.A., Duru C.B., Diwe K.C., Chineke H.N., Merenu I.A., Oluoha U.R., Madubueze U.C., Ndukwu E., Ohale I. (2017). Knowledge, attitude and practices of food hygiene among food vendors in Owerri, Imo State, Nigeria. Occupational Diseases and Environmental.

[52] Singh S., Singh R. (2024). Knowledge attitude and practices of food handlers towards food hygiene. Asian Journal of Agricultural Extension, Economics & Sociology.

[53] Chekol C., Andualem M., Hussien M. (2021). Food safety practice and associated factors among street food vendors in City Administrations of West Gojjam Zone, Northwest Ethiopia, Aus. Food Sci. (N. Y.).

[54] Umar A.A., Jamila T.M. Aliyu U. (2018). The effect of food hygiene training among street food vendors in sabon gari local government area of kaduna state, Nigeria. Sub-Saharan African Journal of Medicine.

[55] Marutha K.J., Paul K.C. (2020). Safe food handling knowledge and practices of street food vendors in Polokwane Central Business District. Foods.

