# Supplementary material for: Food hygienic practices and associated factors among street food vendors in Bishoftu town, central Ethiopia
Source: Heliyon. 2024 Dec 5;10(24):e40938. doi: 10.1016/j.heliyon.2024.e40938 (PMC11665452; doi:10.1016/j.heliyon.2024.e40938)
Supplement: Multimedia component 1 [file mmc1.pdf]

Salale University College of Health Sciences
Chief Academic and Research Vice Directorate

Ref.No C.H.S. 241. 2023
Date June 10, 2023

To: Firaol Girmaa, Tamiru Yazew, Dinaol Bedada, Agama Daba, Chala G. Kuyu

Subject: Providing Ethical Approval

We are pleased to inform you that the research proposal titled "**Food hygienic practices and associated factors among street food vendors in Bishoftu town, central Ethiopia with Reference Number: RRC:No/SLU/ 241/2023**" has been reviewed and approved by the Ethical Review Committee of Salale University College of Health Sciences.

This approval is granted based on the following considerations:

- **Research Significance:** The study addresses crucial aspects of food hygiene practices among street food vendors, which is vital for public health and safety in Bishoftu town.
- **Ethical Compliance:** The proposal has met all ethical standards and guidelines set by the committee, ensuring that the research will be conducted with the highest ethical considerations.
- **Informed Consent:** All participants will be provided with clear and comprehensive information about the study and will give their informed consent prior to participation.
- **Confidentiality:** The confidentiality of all participants will be strictly maintained, and data will be handled with utmost care to protect their privacy.
- **Risk Minimization:** The study design includes measures to minimize any potential risks to participants.

Please ensure that all ethical guidelines are strictly followed throughout the research process. The Ethical Review Committee reserves the right to audit the research activities to ensure compliance with approved protocols.

If you have any questions or require further information, please do not hesitate to contact us.

Sincerely,

Tamiru Yazew Asfaw
Ass't. Professor
Academic and Research V/Director

Salale University
College of Health Science

Phone number ☎ 0111609352 P.B.O. 245 Email: mengistu.tesema@slu.edu.et

CamScanner
